# Nondestructive Detection and Quality Grading System of Walnut Using X-Ray Imaging and Lightweight WKNet

**DOI:** 10.3390/foods14132346

**Published:** 2025-07-01

**Authors:** Xiangpeng Fan, Jianping Zhou

**Affiliations:** 1 Agricultural Information Institute, Chinese Academy of Agricultural Sciences, Beijing 100081, China; 2 Key Laboratory of Agricultural Big Data, Ministry of Agriculture and Rural Affairs, Beijing 100081, China; 3 College of Mechanical Engineering, Xinjiang University, Urumqi 830017, China; linkzhou@163.com; 4 Agriculture and Animal Husbandry Robot and Intelligent Equipment Engineering Research Center of Xinjiang Uygur Autonomous Region, Urumqi 830017, China

**Keywords:** walnut internal quality, nondestructive detection, X-ray imaging, YOLO v5s, lightweight model

## Abstract

The internal quality detection is extremely important. To solve the challenges of walnut quality detection, we presented the first comprehensive investigation of walnut quality detection method using X-ray imaging and deep learning model. An X-ray machine vision system was designed, and a walnut kernel detection (called WKD) dataset was constructed. Then, an effective walnut kernel detection network (called WKNet) was developed by employing Transformer, GhostNet, and criss-cross attention (called CCA) module to the YOLO v5s model, aiming to solve the time consuming and parameter redundancy issues. The WKNet achieved an mAP_0.5 of 0.9869, precision of 0.9779, and recall of 0.9875 for walnut kernel detection. The inference time per image is only 11.9 ms. Extensive comparison experiments with the state-of-the-art (SOTA) deep learning models demonstrated the advanced nature of WKNet. The online test of walnut internal quality detection also shows satisfactory performance. The innovative combination of X-ray imaging and WKNet provide significant implications for walnut quality control.

## 1. Introduction

Walnut (*Juglans regia* L.) is one of the four most important agricultural nut products in the world [1]. It contains a large amount of protein, fat, vitamins, and minerals [2,3]. Numerous studies demonstrated that walnuts had various physiological and pharmacological functions, such as antioxidant [4], anti-diabetes [5], and anti-fatigue properties [6], which makes the walnuts earn the reputation of “longevity fruit”. However, the lack of standardized technical support for walnut growth, production and storage chain management can cause the walnuts’ quality to degrade [7], such as kernel blackening, becoming moldy, and other deterioration [8]. This seriously affects the nutrition and commodity value of walnuts. With the consumers’ increasing demands towards food safety and quality, more green and healthy walnuts are needed [9,10]. The hard shells of walnuts pose great challenges in determining the internal quality [11]. Human labors in walnut processing factories are costly, time-consuming, and are not effective [12]. Thus, it is of great significance to develop a rapid nondestructive quality detection and grading method.

The machine vision technology is able to visually acquire the external features of agricultural products. However, the only visible light imaging inspection technique cannot determine the internal quality of the nuts. Several researchers have tried to detect walnut quality using spectral analysis technology [13], machine vision technology [14] and Terahertz technology [5]. X-ray images, also referred to as radio graphs, make nondestructive visualization for quantification internal structures possible [15], which had been gradually applied in the quality detection of agricultural products [16,17]. Karunakaran et al. proved the potential of soft X-ray methods to detect internal infestations in grain samples [18]. Alves et al. proposed a simple X-ray imaging system for agricultural product quality control [19]. Wu et al. studied the wheat tiller morphological traits detection method based on X-ray tomography technology [20]. Raju et al. utilized an X-ray CT scanner to generate pepper seeds images and performed image classification investigation [21]. Gao et al. integrated X-ray imaging technology and traditional image processing technology for walnut mass detection [22]. Van et al. combined support vector machine with a feature extraction algorithm using X-ray imaging to detect internal flaws of pear fruit [23]. The accuracy of proposed method achieved more than 90.2%. Ma et al. used full spectrum band, RGB spectrum band and total color difference characteristic spectral band as an input, and established a walnut kernel appearance quality classification model using decision tree, K-nearest neighbor, and support vector machine algorithm [24]. Compared to other spectral techniques, X-ray imaging technology has the characteristics of fast detection speed, high precision, low cost, and nondestructive attributes. Furthermore, it has incomparable advantages in internal quality detection of agricultural products with the help of image processing technology. However, the current research using traditional image processing methods suffered from poor performance due to the weak feature extraction ability. Deep learning technology could automatically learn representative features of images with multiple levels of abstraction [25], which could dramatically improve the end-to-end object recognition effects [26]. Deep learning networks have been the frequently established approaches on nondestructive detection in numerous fields [27,28]. You Only Look Once (YOLO) serial models are deep learning-based single stage real-time object detectors that have grown from version to version, and they have shown remarkable performance for agricultural production quality classification [29,30]. Being commonly used object detection methods [31,32], they have demonstrated the high potential and broad applicability in industries, such as quality control, sorting, and packaging. Wang et al. proposed a walnut target detection algorithm based on YOLO v5 to meet the quality detection demand [33].

Based on the above considerations, we propose integrating X-ray imaging technology, machine vision, and deep learning technology to detect walnut internal quality and carry out a grading operation. Machine vision applications often require real-time image acquisition and analysis by various autonomous transmission systems. It remains a significant challenge to implement these object detection algorithms in real world scenarios. Therefore, it is necessary to choose a deep learning model with higher classification accuracy and faster inference speed. The major contributions of this paper are summarized as follows:

1. An X-ray imaging system and a walnut kernel detection (WKD) dataset including 1756 images with 8472 bounding boxes are constructed.
2. A novel rapid and lightweight WKNet is proposed by employing the efficient Transformer, GhostNet, and criss-cross attention (CCA) module to the advanced YOLO v5s deep learning model, aiming to solve the problem of poor feature extraction ability, parameter redundancy, and computing time consuming.
3. A comprehensive investigation ranging from qualitative and quantitative evaluations of WKNet model are carried out to obtain best performance using the self-built WKD dataset.
4. Some insights are given by our evaluation and analysis for walnut internal quality detection by deploying the WKNet to walnut quality control equipment.

## 2. Materials and Methods

### 2.1. The Principle of X-Ray Technology and Images Acquisition

#### 2.1.1. The Principle of X-Ray Imaging Technology

X-ray imaging technology is the physical law of attenuation of X-rays and Radon transform [16]. Photons in a soft X-ray beam are being absorbed, transmitted, or scattered when the photons pass through a sample. And in general, the higher material density and atomic number of constituents are, the greater the X-ray absorption is. The attenuation intensity is indicated by the Beer-Lambert law [20], as shown in Formula (1):

(1) $$I=I_{0}e^{-\mu_{n}L}$$

where *I* is the intensity of X-ray exiting through a sample in eV, *I*_0_ is the intensity of X-ray in eV, *μ_n_* is the linear attenuation coefficient of a sample on the wavelength in eV/mm, and *L* is the path length through a sample in mm. The walnut consists of a wooden shell, a distraction wood and a nucleolus. X-ray imaging technology has different mass attenuation characteristics, which is significant for the different structures comprising walnuts. In an X-ray imaging system, the structure difference in physical density could be visualized through the changes in image intensity.

#### 2.1.2. X-Ray Image Acquisition

**Figure 1:**
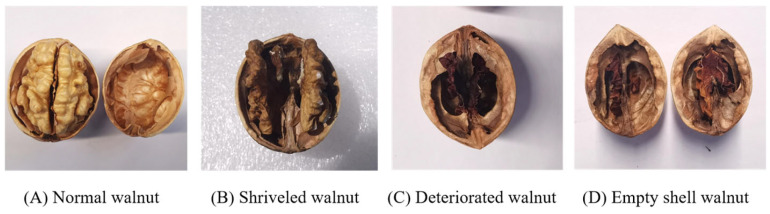
Some examples of walnut kernels.

Walnuts of ‘*Wen 185*’, ‘*Xin 2’*, and ‘*Xinfeng*’ from the Xinjiang Region were used as samples for dataset acquisition. To ensure the accuracy and robustness of the detection model, the Chinese national standard “GB/T 20398-2021 walnut nut quality grade” [34] was used to distinguish different quality types of walnuts. Some examples of the walnuts are shown in Figure 1. The average transverse diameter of walnut is 33.1 mm and the average longitudinal diameter is 40.5 mm. Through the blade cutting and manual natural separation, the walnuts were cut one by one to check the quality of the walnut kernel. Then, the cut walnuts were sealed up and marked as good or bad. The good walnuts are fuller, and their X-ray images have a clearer internal texture. Bad walnut kernels are fragmentary and incomplete, and the X-ray scanned images have noticeable empty shell states. The purpose of this operation is to obtain a correctly labeled dataset and to perform supervised model training. There were in total over 8000 plump, shriveled, deteriorated, and empty shell walnuts.

Different from the general visible image acquisition, the X-ray imaging system generally only has the operation detection under fixed working parameters (tube voltage, tube current, etc.). According to multiple rounds of experimentation, it was found that the image quality was best when the tube voltage and tube current are 45 kV and 6.5 mA, respectively. Automatic acquisition was mainly carried out by linear array detector and the walnut X-ray images were collected. Under fixed working parameters, there were 1756 useful original images collected for model training, validation, and testing. The format of images is PNG, and the resolution of X-ray image is 1025 × 500 pixels.

#### 2.1.3. X-Ray Images Preprocessing

**Figure 2:**
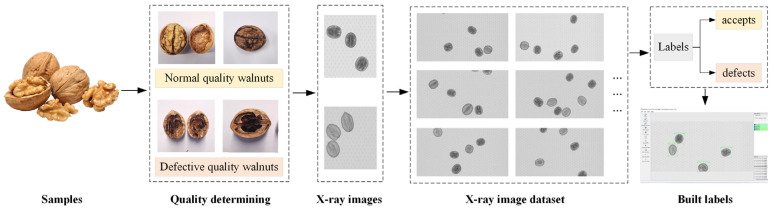
The sketch map of X-ray images labeling.

There are many small gray dots in the background of the X-ray images of the walnuts obtained during the transportation of walnut on the conveyor belt because of the requirement of friction for effective transmission. The existence of gray dots will have a certain influence on image detection. Therefore, some image enhancement processing methodologies were carried out to reduce noise and improve the image quality. Image gray transformation combined with contrast enhancement is the most commonly used method. The gray dots were removed and the contrast of the X-ray walnut image was effectively improved while preserving the necessary details of the image. At the same time, the difficulty of subsequent detection process was reduced. Annotation is another important preprocessing step for supervised training of deep learning models. Two experts in the field of primary food processing standards gave advice on determining plump, shriveled, empty shells of walnuts for annotation. Considering the practical application of a walnut quality detection system, a classification label for the walnut quality was established (Figure 2). Walnuts of good quality are defined as “accepts”, and those bad quality walnuts of shriveled, deteriorated, and empty-shell are defined as “defects”.

### 2.2. The Proposed WKNet

Walnut X-ray images contain multi-level semantic information. This information shows visual attribute traits that can be automatically identified by deep learning based visual perception technology [35]. Thus, how to extract the semantic information is the key to improving walnut internal quality detection effect. The actual scenario of the walnut kernel quality detection is a moving conveyor belt, thus the detection model must have lightweight structure and low detection delay for real-time performance. In this section, we will introduce our proposed WKNet in detail, including the overview and the related modules for meeting the requirement of real-time detection and online grading.

#### 2.2.1. Overview

**Figure 3:**
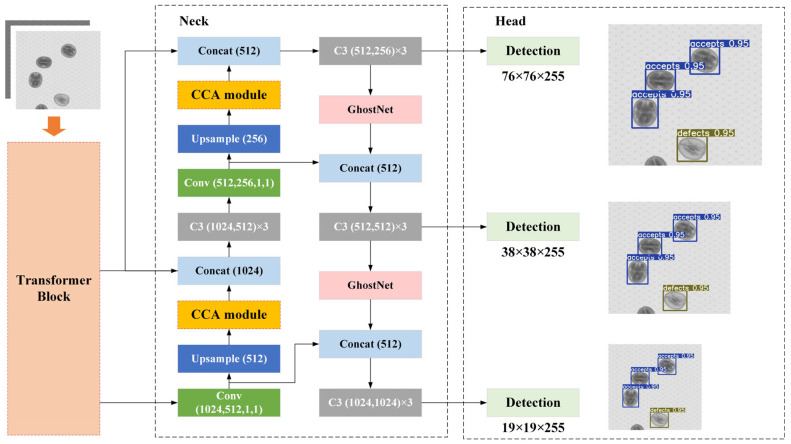
The overall structure of the WKNet model.

YOLO v5 is an efficient one-stage anchor-based objection algorithm, which is modified from the pioneer work of predecessors [36]. It is widely used in target detection scenarios due to its advantages of higher detection accuracy and faster computing speed [37]. The YOLOv5s performed well in the pursuit of a trade-off between accuracy and speed, which could offer the fastest inference speed being up to 140 FPS (frames per second). In order to meet the lightweight requirements, the detection model of YOLO v5s was selected as the basic deep learning architecture. YOLO v5s mainly includes four parts [38]: Input, Backbone, Neck, and Head. The Input layer is mainly responsible for image preprocessing and anchor frame generation. The Backbone extracts feature maps of different levels through convolutional operations. The Neck layer uses feature pyramid network and path aggregation network to enhance the localization feature information. The Head generates the category probability and position information of the target. Though YOLO v5s achieved better performance, the parameters redundancy and huge computation issues limit the promotion of YOLO v5s. Thus, the YOLO v5s model needs to be improved to meet the demands. There are three improvements for YOLO v5s, including the introduction of Transformer structure, the GhostNet module, and the construction of an efficient criss-cross attention module. Transformer block structure has better robustness in the construction process of global information for quality identification. GhostNet makes the model more lightweight. The criss-cross attention (CCA) mechanism is an adaptive resource allocation scheme of the model, which can make the model selectively focus on part of the information. We call the improved YOLO v5s network for walnut kernel detection as WKNet. The overall structure of WKNet is shown in Figure 3.

#### 2.2.2. Transformer Block

**Figure 4:**
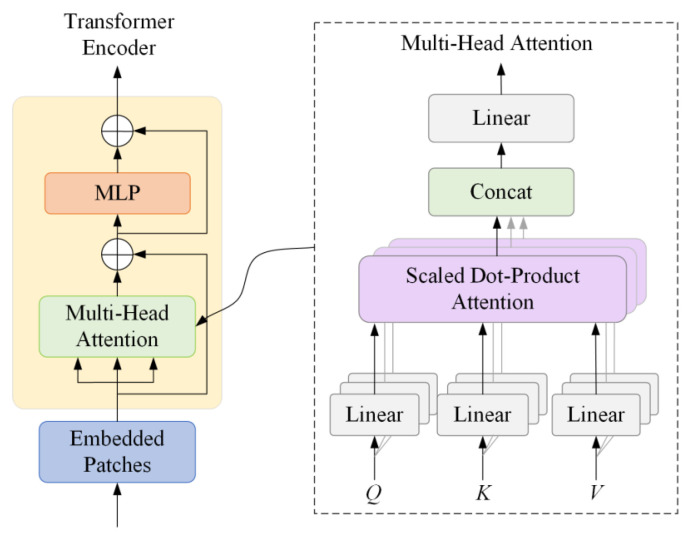
Transformer depth encoder structure diagram.

Transformer deep encoder architecture can capture the global long distance dependencies of the entire feature graph with a self-attention mechanism proposed by Vaswani et al. [39]. It could achieve high recognition accuracy without the use of convolution kernel [40]. In addition, the Transformer structure incorporates learnable location information in the image, which could solve the problem of identifying deviations in traditional CNN, and it has better robustness in the construction process of global information. Thus, the Transformer block structure was utilized to replace the CSPDarknet backbone in the original YOLO v5s. The detailed Transformer encoder structure is shown in Figure 4. It consists of two sub-layers, the first of which consists of multiple attention modules. The second sub-layer is the multi-layer perceptron.

In Transformer encoder structure, embedded patches vector can be mapped into three vectors: query *Q*, key *K*, and value *V*. The similarity between *Q* and *K* vectors can be calculated through dot product. After scaling in a certain proportion and normalization of softmax, the obtained similarity value can be multiplied with *V* value vector to obtain semantic weight. All semantic weights are weighted and summed to obtain self-attention features. Finally, feature graphs with rich global information are obtained by MLP processing. Multi-head attention is a linear projection of the input *Q*, *K*, *V* with a different, learned projection parameter matrix, and then input the dot product attention, repeating the process *h* times. The calculation formulas are shown as follows:

(2) $$A=Attention(Q,K,V)=\mathrm{Softmax}\left(\frac{QK^{T}}{\sqrt{d_{k}}}\right)V$$

(3) $$Multihead=\mathrm{contant}(A_{1},A_{2},\ldots ,A_{j})W$$

(4) $$\mathrm{Softmax}(t)=\frac{e^{t}}{\sum_{j=1}^{N}e^{t}}$$

where *A* is the self-attention feature, *Q* is the image attention query vector, *K* is the image attention key vector, *V* is the image attention value vector, *d_k_* is the scaling factor, representing the dimensions of vector *Q* and vector *K*. In order to obtain the probability distribution over the feature position, the attention calculation uses the Softmax function. The function of Softmax is normalization, which makes the probability assignment more obvious. *Multihead* function simply connects the head outputs of different spaces by uniting multiple independent attention instances together to determine the final attention. *W* represents the linearized coefficient matrix. Contant (*A*_1_, *A*_2_, …*A*_j_) represents different containers of self-attention mechanisms. The major role of self-attention is to solve the problem of contextual interaction and spatial dependence.

#### 2.2.3. GhostNet Makes Model Lightweight

**Figure 5:**
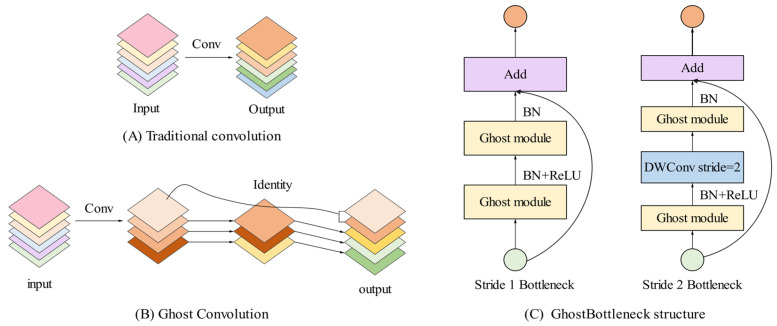
Structure diagram of GhostNet module.

To solve the problem of parameter redundancy, the GhostNet was applied to replace some ordinary convolution block in the YOLO v5s to obtain more lightweight model. The GhostNet was first proposed to generate more feature maps from cheap operations [41]. The structure diagram of GhostNet is shown in Figure 5. Different from traditional convolution blocks, Ghost Convolution performs feature map extraction on images in two steps [42]. The first step is using a small amount of traditional convolution to generate *m* original feature maps. The second step is using *m* original feature map after linear operation to regenerate *s* Ghost feature graph.

Suppose the input channel is *c*, the height and width of the feature graph are *h* and *w*, relatively. The height and width of the output data are *h*′ and *w*′, relatively. The number of convolution kernels is *n*, the size of the convolution kernels is *k*, the size of the linear transformation convolution kernels is *d*, and the number of transformations is *s*. Theoretically, the parameter compression ratio calculation process using Ghost convolution to replace traditional convolution is as follows:

(5) $$r_{c}=\frac{n\cdot c\cdot k\cdot k}{\frac{n}{s}\cdot c\cdot k\cdot k+(s-1)\cdot \frac{n}{s}\cdot d\cdot d}\approx \frac{s\cdot c}{s+c-1}\approx s$$

The calculation formula of the acceleration ratio of the network model is as follows:

(6) $$\begin{array}{l} r_{s}=\frac{n\cdot h^{\prime }\cdot w^{\prime }\cdot c\cdot k\cdot k}{\frac{n}{s}\cdot h^{\prime }\cdot w^{\prime }\cdot c\cdot k\cdot k+(s-1)\cdot \frac{n}{s}\cdot h^{\prime }\cdot w^{\prime }\cdot d\cdot d}\\ =\frac{c\cdot k\cdot k}{\frac{1}{s}\cdot c\cdot k\cdot k+\frac{\left(s-1\right)}{s}\cdot d\cdot d}\approx \frac{s\cdot c}{s+c-1}\approx s \end{array}$$

Through the above formulas, the calculation of acceleration benefits and the effect of parameter compression is affected by the number of transformation, that is, the more Ghost feature maps generated the more lightweight the model is. Thus, the acceleration effect becomes better, and the detection accuracy also declines.

#### 2.2.4. Criss-Cross Attention Mechanism

**Figure 6:**
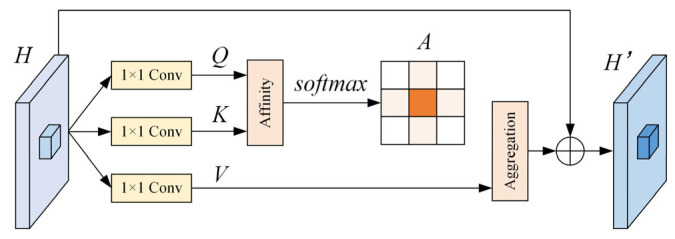
Structure diagram of CCA module.

The attention mechanism can independently learn the semantic information in the images according to degree of importance [43]. Considering light-weight computation and memory conditions in dynamic scenes, we designed the criss-cross attention module to insert in the prediction layer of YOLO v5s. The CCA module collects contextual information in horizontal and vertical directions to enhance pixel-wise representative capability in a very effective and efficient way. It originates from the design of Huang et al. [44], which is adapted to the rapid detection of walnut quality in the operation of walnut X-ray sorting. The structure of CCA module is shown in Figure 6. Based on non-local attention module, it uses twice attention weighting and multiple sparse attention diagrams to replace of single intensive attention structure to save resource consumption.

Specifically, given a local feature map ***H***∈R*^C^*^×*W*×*H*^, the module first applies two convolutional layers with 1 × 1 filters on ***H*** to generate two feature maps ***Q*** and ***K***, respectively, where {***Q***, ***K***}∈R*^C^*^′×*W*×*H*^. *C*′ is the number of channels, which is less than *C* for dimension reduction. After obtaining ***Q*** and ***K***, an attention feature map ***A*** is generated through the Affinity operation and Softmax operation:

(7) $$d_{i,u}=Q_{u}K_{i,u}^{T}$$

where *d_iu_*∈*D* is the degree of correlation between features ***Q****_u_* and ***K****_iu_*, *i* = [1,2,…*H* + *W* − 1], and *D*∈R^(*H+W*−1)×(*W*×*H*)^. ***K****_iu_* is the *i*-th element of ***K****_u_*, which can be obtained by extracting feature vectors from ***K*** which are in the same row or column with position *u*. Then, another convolutional layer with 1 × 1 filter is applied on ***H*** to generate ***V*** for feature adaption. At each position *u* in the spatial dimension of ***V***, we can obtain a vector ***V****_u_* and a set ***M****_u_*∈R^(*H+W*−1)×C^. The contextual information is collected by an Aggregation operation using an attention feature map ***A*** defined as follows:

(8) $${H^{\prime }}_{u}=\sum_{i==0}^{H+W-1}A_{i,u}M_{i,u}+H_{u}$$

where ***H***′*_u_* is a feature vector in ***H***′∈R*^C^*^×*W*×*H*^ at position *u* and ***A****_iu_* is a scalar value at channel *i* and position *u* in ***A***. The contextual information is added to local feature ***H*** to augment the pixel-wise representation. Feature map ***H***′ can selectively combine contextual information according to the spatial attention map, where each position is sparsely connected to other positions in the same row and column. In this paper, two CCA modules are connected to obtain a double-cross-attention module, through which each position of the feature map can perceive the complete context information from all the input pixels.

## 3. Experiment Results and Discussion

### 3.1. WKNet Model Experiment Platform and Evaluation Metrics

**Figure 7:**
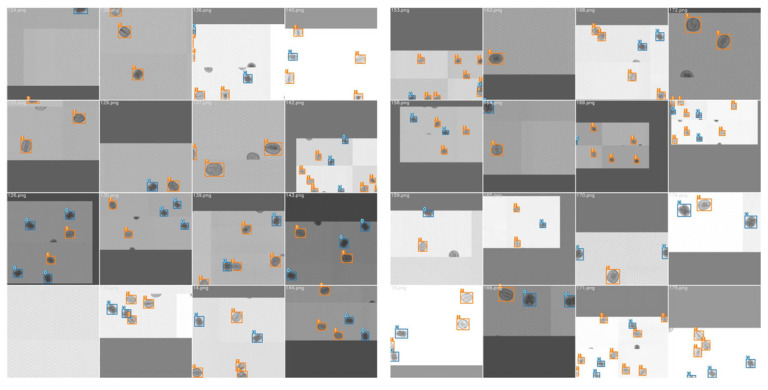
Mosaic enhancement effect of training data.

In this section, we describe the experiment configurations and evaluation metrics. The operation environment of the proposed WKNet is illustrated in Table 1. The GPU was an NVIDIA GeForce RTX 2060, and the Pytorch deep learning framework was built under the Ubuntu 20.04 operating system. The program was written in Python language, and the CUDA version was 11.1. The training set, validation set, and test set were divided as the ratio of 7:2:1. Before training, the dataset was enlarged by eight times to avoid over-fitting using random rotation and geometric transformations. The number of training images was enlarged to 9834, the number of validation images was 2810, and the number of test sets was 1414. The mosaic enhancement was also utilized in the training process. Mosaic enhancement effect is shown in Figure 7. The training was executed using an initial learning rate of 0.01 and a termination learning rate of 0.001. The optimizer momentum was 0.92. The input images were normalized to a resolution of 640 × 640. Each training proceeded for a maximum of 300 epochs, and the batch size was 16.

The evaluation metrics in this experiment include Precision (*P*), Recall (*R*), *F*1, Mean Average Precision at the threshold of 0.5 (mAP_0.5), model size and inference time. *P* is the ratio of true positive detections to the total predicted positives, which measures the proportion that the model predicts correctly. *R* is the ratio of true positive detections to the total actual positives, which can measure the percentage of targets the model predicts correctly. *F*1 is the comprehensive index of *P* and *R*, which balances both metrics to provide a single performance measure for the model. mAP_0.5 measures the overall performance of model accuracy. Inference time (short for Infer time) evaluates the real-time performance of the model. The specific calculation methods of *P*, *R*, *F*1 and mAP_0.5 can be found in reference [45] and reference [27].

### 3.2. WKNet Experiment Results and Analysis

#### 3.2.1. Training and Validation Results of WKNet

**Figure 8:**
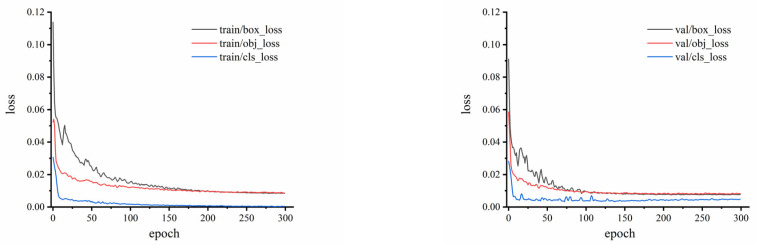
Train and validation loss curve of WKNet.

**Figure 9:**
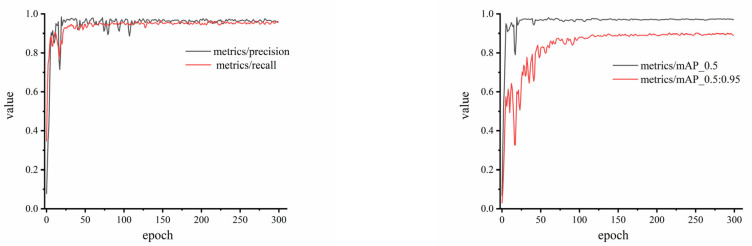
The metrics of WKNet model after training.

In order to more intuitively understand the model training effects, the loss values in the training and verification process are depicted, as shown in Figure 8. The curve of box_loss illustrates the error between the prediction frame and the calibration frame. The curve of obj_loss is utilized to describe the confidence of the model. The curve of cls_loss is used to evaluate whether the calculation anchor frame and the corresponding calibration classification are correct. The smaller the loss, the more accurate performance the model has. The loss curves of training show that the model converges around the epoch of 200, the loss curves of validation also converge quickly at lower loss values. It can be concluded that the convergence speed is fast, the loss value is low, and the training performance is better guaranteed. In Figure 9, the model metrics of trained WKNet are illustrated. The mAP_0.5:0.95 represents the average mAP at different IoU thresholds (from 0.5 to 0.95, step size is 0.05). The precision and recall achieved more than 0.95, with the convergence at about 150 epoch. The mAP_0.5 is relative to 1.0 at the epoch of 100, which means that the whole process is basically steady. It could also be found that the shaking change in mAP_0.5:0.95 is obvious before epoch 100, while the mAP_0.5:0.95 is steady after 180 epoch. The training and validation process of the experiment indicates the high performance of the proposed model.

#### 3.2.2. Walnut Quality Detection Result Based on WKNet

**Figure 10:**
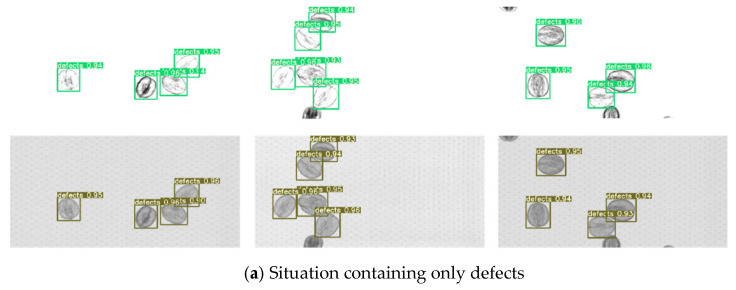

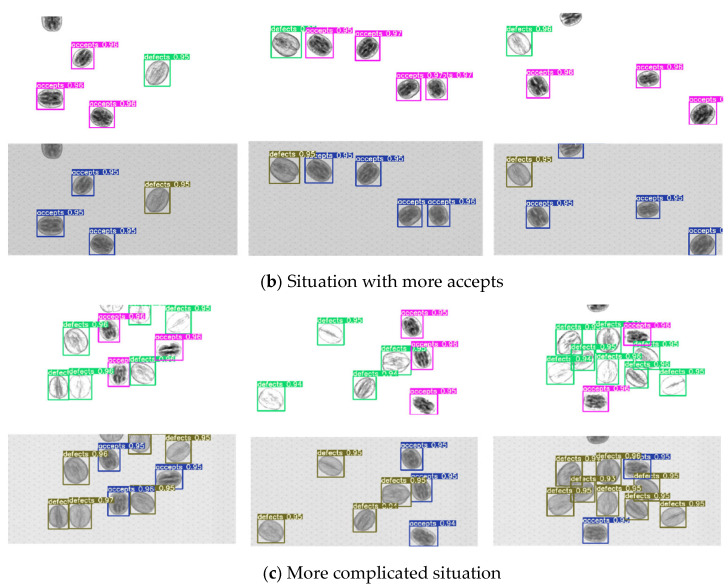
Partial test set visualization results of walnut quality detection.

In this section, the detection effects of test dataset are visualized. One test dataset is the raw images without processing, the other is the images with background removal. Partial visualization results of walnut quality detection are shown in Figure 10. The visual results show that the confidence level of the detected walnuts is above 0.9, indicating that the proposed model has learned the real features of different quality of walnuts, and the recognition effect is satisfactory with high precision boundary boxes. We also conduct five-fold cross-validation experiments to comprehensively evaluate the robustness and generalization of our proposed WKNet.

The obtained mAP_0.5, *P*, *R*, and *F*1 under different dataset attributes are summarized in Table 2. From Table 2, it can be found that the performance metrics of image background removal group are higher than those of images without background treatment. The WKNet model trained on background removal image datasets has a mAP_0.5 of 0.9869, precision of 0.9779, and recall of 0.9875. The inference time of two methods is compared with the model trained on original background images, the mAP_0.5 increased by 0.0153, the *P* increased by 0.0114, and the *R* increased by 0.0275. The inference time per image of the model decreased from 13.4 ms to 11.9 ms. The above experiment results prove that the training effects of WKNet can be effectively improved after background removal processing. Thus, the dataset pretreatment method of removing background by image contrast enhancement is essential for real scenarios. The high performance can fully meet the requirement of rapid and accurate detection for walnut quality grading systems.

#### 3.2.3. Feature Visualization Analysis

**Figure 11:**
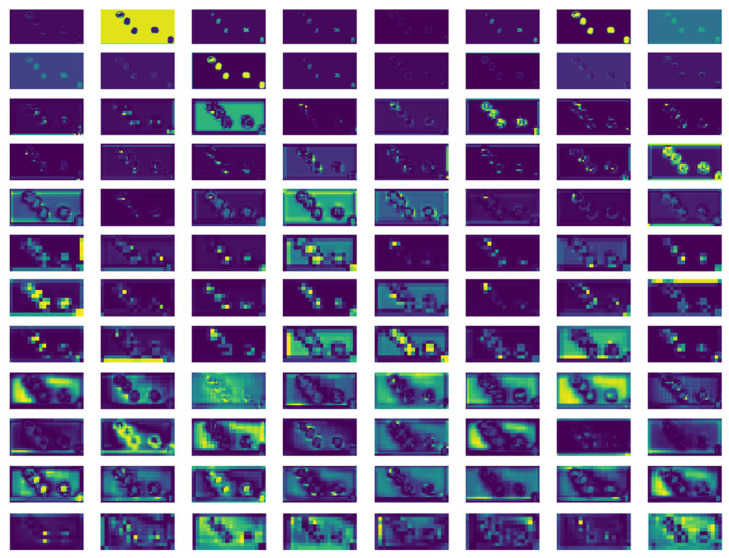
Feature maps of different level convolutions in the walnut quality detection process.

In order to explore how the WKNet model extracts important features of walnut objects, the feature maps of different level convolutions were extracted and saved to the computer. Figure 11 shows the feature maps of different channels in the walnut quality detection process. From top to bottom of Figure 11, the convolutional neural network structure that generates the feature map becomes deeper and deeper, that is, closer and closer to the output layer of WKNet. The initial feature maps output retain almost all the original information of the walnut image. As the number of network layers increases, the feature becomes more and more abstract when the number of layers deepens. There is less concrete information about the walnuts of the image, but more information about the category. In the whole prediction process, the model can always focus on the important walnut targets, which can help us better understand the convolution and sampling mechanism of the model.

#### 3.2.4. Ablation Experiments of WKNet

In order to verify the effectiveness of the three improvement strategies in YOLO v5s, eight group ablation experiments were carried out. In these experiments, the model hyper-parameters were unified, comparison, and ablation experiments were performed on the same dataset and the same experiment platform. The ablation experiment results are shown in Table 3. Different improvement strategies improved the performance of the original YOLO v5s model to varying degrees. The Transformer module could enhance the accuracy and real-time performance. The GhostNet structure reduced the inference time to the fullest extent, while the mAP_0.5 is relative to that of Transformer block. The application of the CCA module not only reduces the number of model parameters, but also better extracts the depth characteristic information of different walnuts and improves the detection accuracy. Therefore, the lightweight convolution method GhostNet can also extract effective training features. It is proved that the effective feature layer is not necessarily obtained through the complete convolution of multiple layers, and the same effect can be achieved by efficient convolution methods including Transformer and GhostNet. The combination of Transformer and GhostNet enhances the mAP_0.5 to a greater extent. It also indicates that the GhostNet structure contributes more to reducing the inference time of walnut images than Transformer structure. However, the combination of Transformer, GhostNet, and CCA could sharply improve the model performance significantly, including mAP_0.5 and detection speed.

#### 3.2.5. Comparison Experiments to SOTA Methods

In this section, we further compared the performance between our proposed WKNet and the state-of-the-art (SOTA) models. The experimental results are shown in Table 4. It can be seen that the two-stage model Faster R-CNN costs much more time for inference than one-stage models. YOLO v5 serials model could achieve higher mAP_0.5 and *F*1 values, as well as the shorter inference time. The mAP_0.5 increases with the deepening of network depth. However, the YOLO v5x has the lower mAP_0.5 and *F*1 score than YOLO v5m. The possible reason may be that the model is too complex, and the data are simple, which leads to the over-fitting in the training process. As for inference time, YOLO v5s has better performance than YOLO v5m, YOLO v5l, YOLO v5x, YOLO v6, YOLO v7, YOLO v8, YOLO v9, and Real-time detection transformer (RT-DETR). Meanwhile, YOLO v8n achieved a good balance in mAP_0.5 and inference time. The structure of YOLO v8 is more complex. YOLO v5s model provides a competitive advantage in terms of reliability for quality detection tasks with low complexity. And that is why we chose YOLO v5s as the basic deep learning framework to modify. From Table 4, it can also be found that the mAP_0.5 value of WKNet is the highest, and the inference time is the lowest. The computational complexity of the model is greatly reduced after improvement. Our WKNet outperforms all the SOTA object detection models, which proves that the WKNet is the most applicable model for the walnut internal quality detection.

### 3.3. Walnut On-Line Detection and Quality Grading Test

#### 3.3.1. Walnut On-Line Detection and Grading System Structure

**Figure 12:**
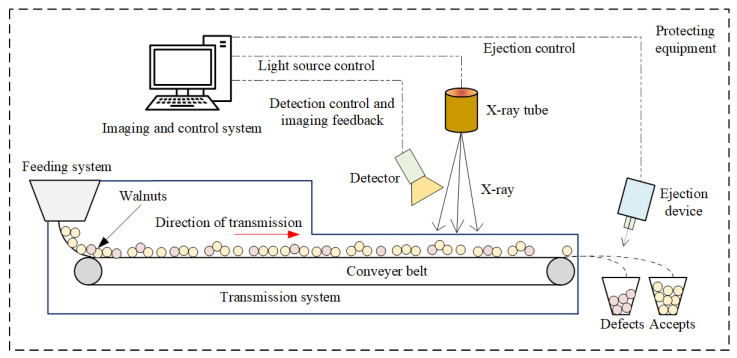
The X-ray imaging and walnut detection and grading system structure.

To further verify the effectiveness of the proposed method in real scenarios, the online walnut quality control experiment was conducted using X-ray imaging and WKNet models. The overall system architecture is shown in Figure 12. The system contains X-ray tube, detector, transmission system, protecting equipment, ejection device, and visual imaging and control system. The image processing system is the core of walnut quality detection with high precision and high speed. The ejection device is an important part of walnut grading accurately after quality detection. The imaging and control system controls the solenoid valve type air blowing device through the drive board, and removes unqualified walnuts detected through the air nozzle.

**Figure 13:**
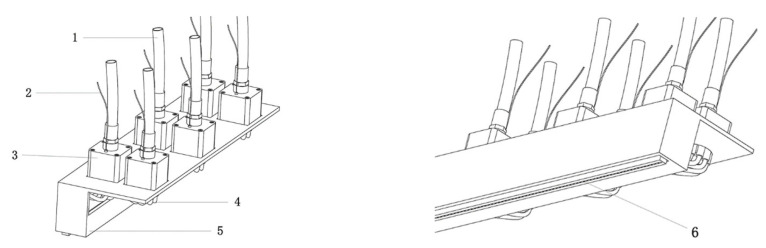
The novel designed air mouthpiece structure used for defected walnut ejection. 1. Air pressure conveying tube ①, 2. driver board connection cable, 3. high frequency solenoid valve, 4. air pressure conveying tube ②, 5. steel plate frame, 6. rectangular air mouthpiece.

TXR-B3-4010BD2-NH72 X-ray detector was used as the test detection host machine, and electromagnetic vibration type was used as the feeding device. In order to improve the ejection effect of defective walnuts, a 72-way air-blown ejection device with a row distributed solenoid valve is designed and equipped in this paper, as shown in Figure 13. The external air pressure production equipment is connected to the air pressure conveying tube ① to the Matrix 890 series high-frequency electric valve, and then the driver plate connection line on the solenoid valve is connected to the driver plate. The computer controller sends instructions to the driver plate to control the closure of the solenoid valve, and the air pressure through the solenoid valve and then through the air pressure conveying tube ② to the air blowing mold. Finally, the air flow is sprayed onto the corresponding walnut through the 5 mm × 1.5 mm miniature rectangular air mouthpiece. The collection device includes a defective walnut collection box and an acceptive walnut collection box. In the design scheme of the removal device, the micro-air vents are distributed in 1 row and 72 columns, so that the walnuts at different positions can be accurately removed by the air flow in the form of flat beam.

#### 3.3.2. Working Parameters and Evaluation Metrics

**Figure 14:**
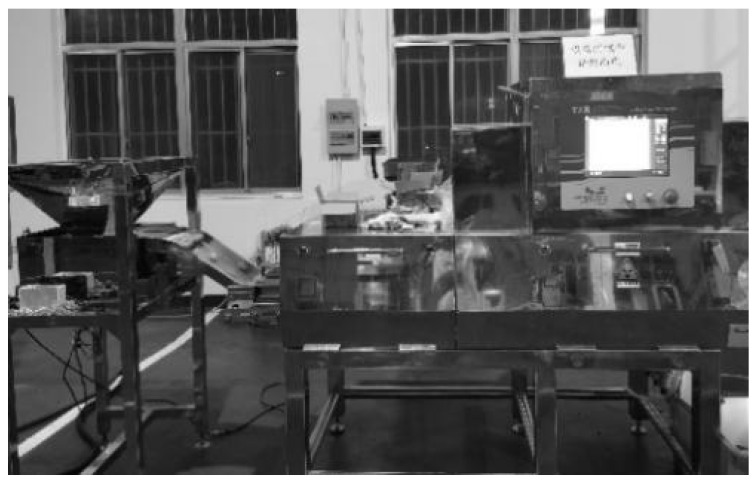
The X-ray imaging and walnut grading system.

The grading working machine is shown in Figure 14. When the technical parameters of core components such as the X-ray tube and detector meet the requirements and are fixed, tube voltage and tube current are the two most important factors affecting the X-ray imaging quality. The tube voltage and current are set at 45 kv and 6.5 mA, respectively. The transmission speed, air blowing delay time, and air blowing duration all have great influence on the ejection results. According to BBD (Box-Benhnken Design) combination-design theory, multi-factor optimization analysis was carried out, and the optimal working parameters of the X-ray detector in walnut were obtained. In this experiment, the transmission speed is 97.00 m/min, air blowing delay time is 135.0 ms, and air blowing duration is 19.00 ms.

In this experiment, the ejection precision φ and error-sorting rate *Y* are selected as the performance evaluation metrics of the quality detection method and the equipment established in this paper. Ejection precision refers to the proportion of qualified walnuts in the acceptance recycling box after all walnuts are detected through X-ray imaging system. The take-sorting ratio refers to the ratio between the defected walnuts and the qualified walnuts by air nozzle after X-ray detection in the defect box. After the walnut X-ray quality control system operated normally for half an hour, samples were collected from the discharge port of the qualified walnut three times, once every ten minutes, with no less than 500 walnuts each time. Qualified and defective walnuts were sorted, respectively, and the ejection precision of three times calculated according to Formula (9) was recorded, and the average value was taken.

(9) $$\varphi =\frac{W-W_{1}}{W}\times 100\%$$

where φ is the ejection precision, *W* is the number of walnut samples collected from acceptance box, *W*_1_ is the number of defected walnut samples from acceptance box. When the walnut X-ray quality control system operated normally for half an hour with walnut X-ray detection, walnut samples were collected from the discharge port of qualified walnut once every ten minutes. There were a total of three collections, with no less than 500 walnuts each time. The quantity of defective walnuts and normal qualified walnuts were recorded, respectively, and the take-sorting ratio was obtained according to Formula (10).

(10) Y=M:m

where *Y* is the take-sorting ratio, *M* is the defected number of walnut samples, *m* is the qualified number of walnut samples.

#### 3.3.3. The On-Line Detection and Grading Test Result

It is of great significance to carry out rapid, non-destructive detection of internal quality during postharvest transport and storage for food safety monitoring and the healthy development of the walnut industry. Based on the proposed X-ray imaging technology and WKNet machine perception model, the walnut sorting experiment was carried out and the test results were obtained (Table 5). The take-sorting ratio depicts that the walnuts on-line qualification detection is essential since the walnut samples vary in quality. High ejection precision metrics prove that the proposed method has satisfactory performance in practical applications and can be used for the real-time on-line qualification detection and sorting of walnuts. The above experimental results fully prove the effectiveness and advancement for walnut quality detection and product grading.

## 4. Discussion

In this paper, we developed a nondestructive technique for walnut quality detection using potential X-ray imaging technology and an advanced deep learning algorithm. The imaging characteristics of walnut were firstly analyzed. Then, we constructed an X-ray-based WKD dataset for deep learning model training and proposed a novel WKNet model to solve the problem of weak feature extraction ability and complex computation. The Transformer block, GhostNet structure, and CCA module contributed a lot to the comprehensive performance of WKNet model. The proposed WKNet reached an mAP_0.5 of 0.9869. The inference time of the model for each image was only 11.9 ms, the parameters and model size were reduced by 56.33% and 57.04%, respectively. The addition of Transformer module, GhostNet, and CCA modules makes YOLO v5s model more lightweight and faster, which is a more suitable walnut quality control system. The walnut on-line detection and quality grading experiment shows satisfactory results for real scenario application. This investigation demonstrated the transformative potential of integrating X-ray imaging, computer vision and deep learning into walnut internal quality. The integration of these enhanced technologies into real-world walnut quality control and grading operations has also shown promising results in industry trials, underscoring their potential for significantly improving food safety control systems. Although the quality detection system we designed outperforms existing algorithms in terms of speed and accuracy, it also has limitations. The model was not sensitive enough to incomplete walnuts in the field of vision. In the future, we still need to further improve the efficiency of the grading system for optimizing the commercial processing performance of walnut.

## Figures and Tables

**Table 1:** The environment configuration of WKNet.

Environmental Attribute	Environment Configuration
Operation system	Ubuntu 20.04
CPU	Intel(R) Core (TM) i7-9750H
GPU	RTX 2060
Memory	32G DDR4
Programs language	Python 3.8.5

**Table 2:** Performance metrics of WKNet using different preprocessing methods for datasets.

Dataset Attribute	Performance Metrics Statistics				
	mAP_0.5	*P*	*R*	*F*1	Infer Time (ms)
Original background	0.9716	0.9665	0.9600	0.9632	13.4
Removing background	0.9869	0.9779	0.9875	0.9827	11.9
Value changing	+0.0153	+0.0114	+0.0275	+0.0195	−1.5

Notes: Infer is short for inference.

**Table 3:** Comparison of test results between YOLO v5s and our WKNet.

Model Type	mAP_0.5	*P*	*R*	*F*1	Infer Time (ms)
YOLO v5s	0.9349	0.9412	0.9283	0.9347	26.2
YOLO v5s (Trans)	0.9613	0.9647	0.9618	0.9624	21.4
YOLO v5s (Ghost)	0.9625	0.9651	0.9622	0.9636	20.6
YOLO v5s (CCA)	0.9627	0.9649	0.9631	0.9640	22.1
YOLO v5s (Ghost + CCA)	0.9774	0.9782	0.9746	0.9764	16.5
YOLO v5s (Trans + Ghost)	0.9669	0.9673	0.9658	0.9665	18.7
YOLO v5s (Trans + CCA)	0.9742	0.9727	0.9719	0.9723	20.3
WKNet (Trans + Ghost + CCA)	0.9869	0.9779	0.9875	0.9827	11.9

Notes: The parameters after YOLOv5s indicates the modules added on the YOLO v5s. Trans is short for Transformer block, Ghost is the GhostNet for abbreviation.

**Table 4:** Comparison of test results between SOTA models and our WKNet.

Model	mAP_0.5	*F*1	Inference Time/ms	Parameters/M	Model Size/M	FLOPs/G
SSD	0.8172	0.8113	274.7	34.4	69.1	98.7
Faster R-CNN	0.9247	0.9233	362.3	137.2	245.7	N/A
YOLO v4-tiny	0.9108	0.8972	37.8	52.4	108.6	216.4
YOLO v5s	0.9349	0.9347	26.2	7.1	14.2	16.3
YOLO v5m	0.9558	0.9471	39.4	21.1	40.6	59.3
YOLO v5l	0.9774	0.9785	44.9	46.6	89.2	171.8
YOLO v5x	0.9316	0.9264	76.1	87.3	166.9	241.9
YOLO v6	0.9322	0.9247	56.7	15.4	20.3	36.8
YOLO v7	0.9418	0.9252	54.5	37.9	72.8	106.1
YOLO v8n	0.9459	0.9328	18.1	3.3	6.3	8.7
YOLO v8s	0.9531	0.9436	34.4	11.2	22.6	28.4
YOLO v9s	0.9627	0.9549	21.8	7.3	16.1	27.6
RT-DETR	0.9562	0.9477	15.6	41.7	81.4	27.2
YOLO v10s	0.9578	0.9556	13.4	7.2	14.3	21.7
Our WKNet	0.9869	0.9827	11.9	3.1	6.1	6.9

**Table 5:** The test result of the walnut qualification detection and sorting experiment.

Walnut Variety.	Ejection Precision φ (%)	Take-Sorting Ratio (%)
Wen 185	98.50	53.96
Xin 2	96.81	46.5
Xinfeng	98.34	53.95
Three mixed varieties	96.65	49.47

## Data Availability

The data are available from the corresponding author.
